# Inhibition of high glucose-induced cardiac fibroblast activation: an effective treatment for diabetic cardiomyopathy using Chinese herbal medicine

**DOI:** 10.3389/fphar.2025.1523014

**Published:** 2025-01-27

**Authors:** Bin Wan, Jing Hu, Yue Luo, Yutong Han, Yaowen Zhang, Qinchuan Huang, Yulin Leng, Chunguang Xie

**Affiliations:** ^1^ Chengdu University of Traditional Chinese Medicine, Chengdu, China; ^2^ Department of Endocrinology, Hospital of Chengdu University of Traditional Chinese Medicine, Chengdu, China; ^3^ TCM Regulating Metabolic Diseases Key Laboratory of Sichuan Province, Hospital of Chengdu University of Traditional Chinese Medicine, Chengdu, China

**Keywords:** diabetic cardiomyopathy, Chinese herbal medicine, myocardial fibrosis, cardiac fibroblasts, mechanism of action

## Abstract

Diabetic cardiomyopathy (DCM) is one of the common diabetic microangiopathy in clinical practice. In the early stage of the disease, there are no obvious clinical symptoms. In the middle and late stages, MF, arrhythmia, and even heart failure may occur, affecting the life and health of patients. MF, as one of the pathological features of DCM at the end stage, is the key factor of poor prognosis leading to ventricular wall stiffness and heart failure, which affects the clinical process and outcome of patients. The development of MF in a high glucose environment involves multiple complex fibrogenic pathways that work together to activate fibroblasts, thereby promoting MF. Indeed, aberrant activation of cardiac fibroblasts (CFs) is a key factor in MF. Therefore, inhibiting the activation of CFs may become a new strategy for the treatment of DCM. Previous studies have shown that Chinese herbal medicine (CHM) has potential in the treatment of DCM. In this review, we first introduced the physiology and function of CFs and discussed the conditions for the pathological activation of CFs in the process of diabetes, and then systematically summarized the effects of CHM on the activation of CFs by controlling the production of advanced glycosylation end products, oxidative stress and inflammation. This review will illustrate the potential of CHM to inhibit the activation of CFs and provide new ideas for the treatment of DCM.

## 1 Introduction

Globally, the prevalence of diabetes is increasing. According to the latest estimates provided by the International Diabetes Federation, by the year 2045, nearly 800 million people are expected to suffer from diabetes globally (International Diabetes Federation, 2021). The prevalence of diabetic cardiomyopathy (DCM) is increasing with the increase in prevalence of diabetes. A typical outcome of diabetes is DCM. It is a complication directly caused by diabetes, independent of coronary artery disease, hypertensive cardiovascular disease, or other heart conditions. It is distinguished by remodeling of the myocardial structure and function (Quaiyoom and Kumar, 2024). In the early stage, the disease is relatively insidious and has no obvious clinical symptoms, while in the middle and late stages, myocardial fibrosis (MF), arrhythmia, and even heart failure may occur (Dillmann, 2019). The first description of this phenomenon was given by Rubler in 1972, who reported on four diabetic patients showing symptoms of heart failure without findings of coronary or valular disease in *post mortem* pathological examinations (Rubler et al., 1972). The epidemiology of DCM shows that the rate of heart failure in diabetic individuals is significantly higher than non-diabetic patients, and diabetes is one of the main causes of cardiovascular disease death, which seriously threatens the health of patients and brings an economic burden to the healthcare system and society (Park, 2021).

So far, there is no particular therapy for DCM. Current standard biomedical treatment for this disease mainly focuses on controlling glucose, strengthening heart diuresis, and nourishing myocardium. Some diabetes medications, such as sodium-glucose cotransporter two inhibitors and glucagon-like peptide-1 receptor agonists, have been shown in studies to be effective in treating DCM (Kato and Kimura, 2020; Marso et al., 2016). Other therapeutic drugs include angiotensin receptor-enkephalin inhibitors and beta blockers (Cohn et al., 2001) (Enzan et al., 2021). These drugs target different pathologic mechanisms to reduce the risk of hospitalization for heart failure and the occurrence of cardiovascular events, while improving heart function. However, the clinical control of the development of DCM in diabetic patients is still not optimistic. So, there is still a long way to go to study the pathogenesis and prevention of DCM.

MF is defined as a myocardial interstitial remodeling process characterized by abnormal growth of cardiac interstitial fibroblasts and excessive accumulation of collagen fibers in myocardial tissue (Gyöngyösi et al., 2017). It is a variety of cardiovascular and related diseases to develop to a certain stage of common pathological changes (Espeland et al., 2018a). According to existing studies, the formation of MF is mainly due to the excessive proliferation of cardiac fibroblasts (CFs) and their transformation into myofibroblasts, resulting in large amounts of extracellular matrix (ECM) deposition in myocardial interstitium, and thus causing cardiac remodeling (Travers et al., 2016; Espeland et al., 2018b; Zhang et al., 2023). As one of the pathological features of DCM that develops to the terminal stage, MF is a key factor leading to ventricular wall stiffness and heart failure due to poor prognosis of the disease, and affects the clinical course and outcome of DCM patients (Tuleta and Frangogiannis, 2021). Therefore, early control of MF progression is critical for preventing or delaying the development of DCM to heart failure.

There is no description about diabetic MF in ancient books of Chinese medicine and modern Chinese medicine generally divides it into diseases such as “chest obstruction”, “heart distension” or “disease accumulation”, and its pathological change involves many aspects, including deficiency of qi, blood stasis and phlegm obstruction (Guanming et al., 2024). In recent years, under the guidance of the theory of traditional Chinese medicine (TCM), the method of treating DCM with CHM has been widely concerned. More and more studies have found that Chinese herbs have potential anti-fibrosis effects, which can objectively and effectively prevent the development of DCM (Ren et al., 2022). CHM refers to plants, animals and minerals derived from nature, which are used to prevent and treat diseases through specific processing (Liu et al., 2013).

At the beginning of the article, we first introduced CFs, along with their physiology and functions, as well as the pathological activation process of CFs in DCM. Subsequently, we utilized multiple databases, including PubMed, Web of Science and China National Knowledge Infrastructure to search for literature on CHM’s inhibition of cardiac fibroblast activation under high-glucose conditions, ensuring a broad coverage of international literature. During the search process, to ensure that only literature directly related to our research question was included, we employed specific keywords such as “CHM”, “cardiac fibroblasts” and “high-glucose condition” and applied Boolean logical operators (AND, OR, NOT) to refine the search results. Finally, we summarized and synthesized the searched literature, aiming to provide new treatment options for the management of DCM.

## 2 Cardiac fibroblasts

### 2.1 Physiological function of cardiac fibroblasts

**FIGURE 1:**
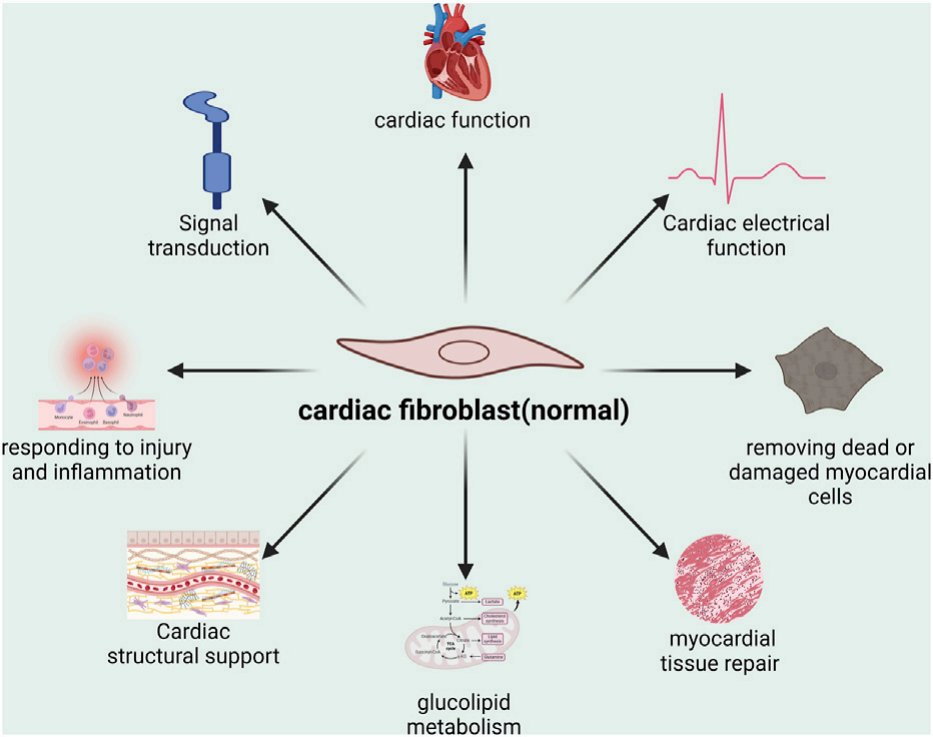
Physiological function of cardiac fibroblasts under normal conditions.

During the process of heart development, the majority of Cardiac Fibroblasts (CFs) originate from the epicardium during the embryonic stage, while a portion of them derive from the endocardium (Wang Y. et al., 2020). They are one of the most common cell types in the adult heart and play a significant physiological role within it. Normally, CFs synthesizes and secretes collagen, elastin and other ECM that provide structural support for the heart (Pesce et al., 2023). It also regulates the function of cardiomyocytes by secreting cytokines and growth factors. When the heart is damaged, CFs can also migrate to the damaged area to participate in the repair process (Tallquist and Molkentin, 2017). In addition, CFs can sense and respond to changes in mechanical forces and participate in mechanical signal transduction of the heart (Tallquist and Molkentin, 2017). Under physiological conditions, when the heart is stimulated by inflammation and injury, fibroblasts are temporarily activated to myofibroblasts, which can promote the recovery of acute tissue injury (Younesi et al., 2024). Generally speaking, in a healthy heart, CFs have a key role in the structure, function, repair, and signaling of the heart (as shown in Figure 1).

### 2.2 Pathologic activation of cardiac fibroblasts

Myofibroblasts are fibroblasts with smooth muscle cell characteristics, which are characterized by significant contractility, the expression of α-smooth muscle actin (α-SMA), and the secretion of collagen and other ECM protein components. It plays an important role in the repair of damaged tissues and pathological fibrosis (Schuster et al., 2023). The α-SMA mentioned here is a specific marker that promotes the activation of myofibroblasts (Shinde et al., 2017). However, it is worth noting that in pathological tissue repair, the continuous activation of myofibroblasts produces excessive ECM, resulting in abnormal deposition of ECM, which may lead to fibrotic diseases, such as cardiac fibrosis, causing hardening and dysfunction of heart tissue, and ultimately organ failure (Schuster et al., 2023).

In MF, the process of transdifferentiation of CFs into myofibroblasts involves several key factors. It is worth mentioning that the CFs discussed in the process of MF mainly originate from the adult’s inherent fibroblasts. First, the involvement of TGF-β1 is very important (Desmoulière et al., 1993). TGF-β is one of the major cytokines inducing fibroblasts to transdifferentiate into myofibroblasts. It stimulates the Smad signaling pathway by binding to cell surface receptors, enhances alpha-SMA expression, and leads to cardiomyocyte apoptosis, myofibroblast overproduction, and cardiomyocyte ECM deposition in the diabetic heart (Meng et al., 2023). Secondly, heart disease often involves changes in mechanical stress, such as the pull tension after hypertension or myocardial injury, which can promote the transdifferentiation of fibroblasts (van Putten et al., 2016). The induction of cardiomyocytes to mechanical stress is realized through the mechanotransduction of ECM tension by integrin receptors (activating Hippo signal transduction pathway and its main downstream effectors YAP and TAZ) (Mia et al., 2022). Integrin also regulates the activation and transdifferentiation of myocardial fibroblasts by mediating the activation of TGF-β1, and influences the development of MF (Li and Frangogiannis, 2022). In addition, in the pathological process of CFs activation and MF, endogenous composition changes were observed and recorded, while induction of various growth factors and cytokines (such as TGF-β), inflammatory signaling pathways and inflammatory factors (such as TNF-α, IL-1) and upregulation of reactive oxygen species (ROS) were observed and recorded (Li et al., 2023; Frangogiannis, 2022; Kurose and Mangmool, 2016; Liu et al., 2023). They all play an important role in the development of MF. Therefore, understanding the activation conditions of CFs transdifferentiation is essential for the development of new therapeutic approaches.

## 3 Effects of high glucose on cardiac fibroblasts

**FIGURE 2:**
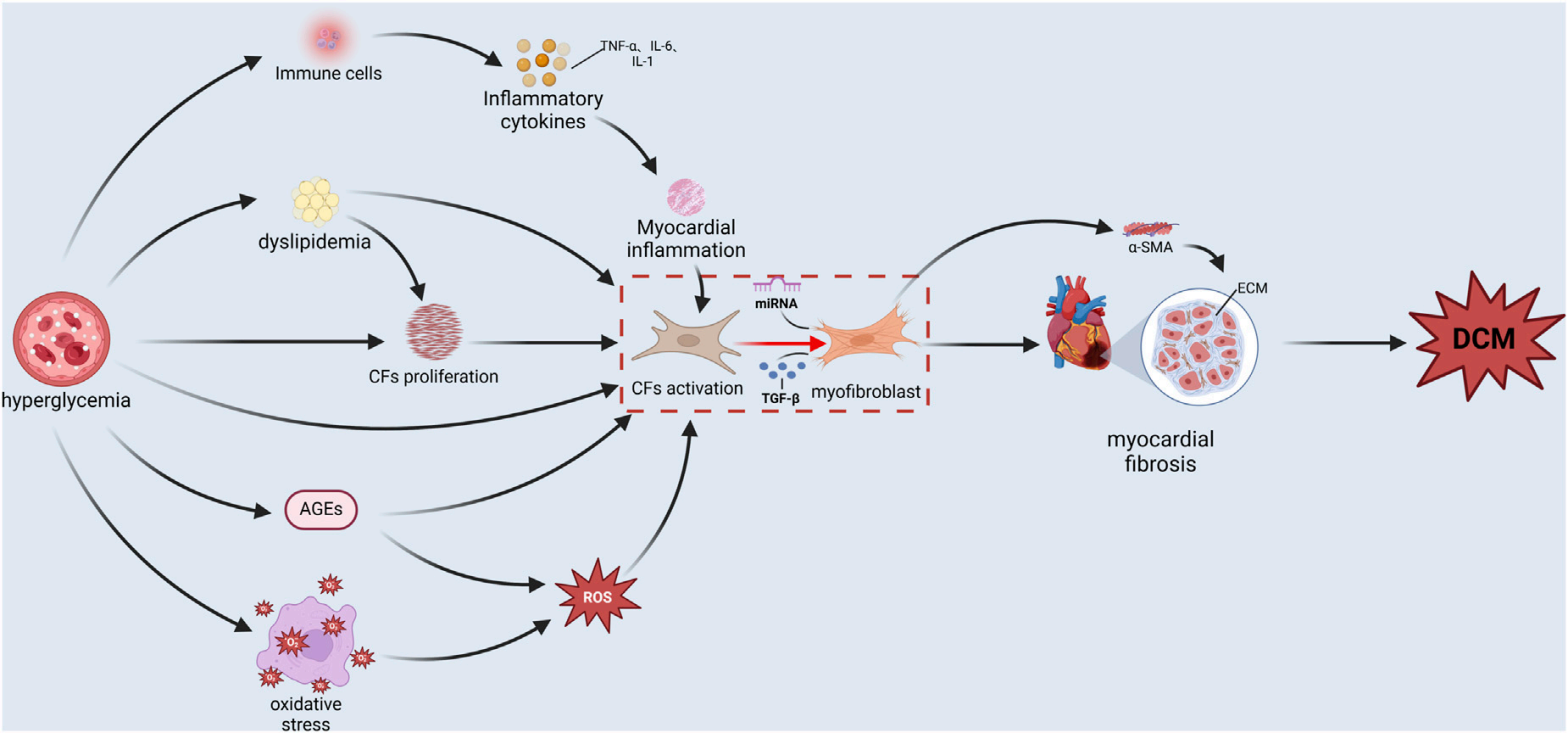
The mechanism of action that stimulates cardiac fibroblast activation under hyperglycemia conditions.

Persistent hyperglycemia, a hallmark of diabetes, is a principal etiological factor that triggers a cascade of pathological processes. These processes, either directly or indirectly, and potentially through interaction can activate CFs, induce MF, and significantly influence the end-stage outcome of DCM (as shown in Figure 2).

### 3.1 Persistent hyperglycemia

Long-term hyperglycemia has a toxic effect on cardiomyocytes, leading to cardiomyocyte injury and the abnormal expression of CFs, eventually increasing the risk of MF (Alex et al., 2023). From established mature rat models of type 2 diabetes, we find that high glucose upregulated TGF-β gene transcription, which induces the expression of myofibroblast phenotype in CFs, while such expressed fibroblast increased collagen synthesis and deposition and promoted the occurrence of fibrosis (Fowlkes et al., 2013). Other studies from a mechanistic standpoint have also demonstrated that high glucose increases the activity of p300-a transcriptional coregulator with intrinsic lysine acetyltransferase activity-and Smad acetylation, thus enhancing TGF-β activity, leading to transdifferentiation of CFs and subsequent fibrosis, hypertrophy, and impaired diastolic function (Bugyei-Twum et al., 2014). High glucose can also activate the Rho/ROCK signaling pathway (a pathway that promotes cell growth, transformation, cytoskeletal adjustment and other cellular processes), induce the expression of visceral adipose hormone and type I procollagen in fibroblasts, and cause excessive proliferation of myoblasts and induce DCM (Hartmann et al., 2016).

### 3.2 Accumulation of advanced glycosylation end products

Hyperglycemia and glucose glycotoxicity can induce protein glycosylation reactions, leading to the increase of advanced glycation end products (AGEs). AGEs are the final products of the combination of excess glucose and protein, which can combine with the body’s tissues and cells and destroy them (Twarda-Clapa et al., 2022). A small amount of AGEs is produced in healthy people, and AGEs accumulate in large quantities in chronic diseases such as diabetes and its complications, Alzheimer’s disease, and atherosclerosis (Twarda-Clapa et al., 2022). Studies have shown that AGEs can activate downstream signaling pathways such as ERK1/2 and PKC-ζ by binding to their receptors RAGE, thereby promoting the proliferation and migration of CFs and triggering the activation mechanism of CFs (Burr and Stewart, 2020; Burr et al., 2020). This enhanced migration of cells and activation of cells can lead to aggravated cardiac fibrosis. Moreover, AGEs can also induce aging of CFs and promote accumulation of the ECM through activating the TGF-β1/Smad signal pathway, resulting in MF (Fang et al., 2016).

### 3.3 Disorder of lipid metabolism

Obesity and insulin resistance, in the environment of type 2 diabetes, lead to the excessive oxidation of fatty acids, further causing abnormal lipid metabolism and accumulation of TG and FFA in cardiomyocytes (Ke et al., 2023). Therefore, large fat deposits and lipid toxicity of lipid droplets can be observed on diseased cardiomyocytes (Nakamura et al., 2022). Abnormal lipid metabolism, such as increased oxidation of fatty acids, is also closely related to CF activation and cardiac fibrosis. In the process of cardiac fibrosis, the reprogramming of lipid metabolism plays an important role. Such metabolic changes not only affect the function of cardiomyocytes, but also affect the behavior of CF and promote the migration and proliferation of CF (Lin et al., 2024). Recent research suggests that in the case of hyperlipidemia, the characteristics of CFs and the origin of different subtypes of fibroblasts may change. In particular, CD34^+^ cells of non-bone marrow origin can differentiate into FABP4+ fibroblasts, which accelerate lipid accumulation through the PPARγ/Akt/Gsk3β pathway, thus promoting the occurrence and development of fibrosis (Du et al., 2024). All of the above studies suggested that abnormal changes in lipid metabolism promote MF by affecting the proliferation of CFs.

### 3.4 Oxidative stress caused by overproduction of reactive oxygen species

Oxidative stress (OS) is a situation where the amount of the active oxygen species generated by the cells in the body surpasses the cells’ antioxidant capacity. Studies have shown that abnormal glucose and lipid metabolism, mitochondrial dysfunction and age accumulation in diabetes can lead to ROS accumulation, aggravate OS, damage cardiomyocytes, activate fibroblasts, promote the fibrosis process, and accelerate the onset of DCM (Peng et al., 2022). ROS promotes the transdifferentiation of fibroblasts into myofibroblasts by activating TGF-β and thus promotes the occurrence of fibrosis. Studies have also found that ROS affects the synthesis, secretion, extracellular self-assembly and cross-linking of ECM components, as well as the degradation of ECM components, thus leading to excess ECM deposition in fibrotic tissues and causing MF (Grosche et al., 2018). Other studies have shown that during myocardial infarction, CFs and mesenchymal progenitor cells are hypoxic, release a large amount of HIF-1α, and produce a high level of ROS (Janbandhu et al., 2022). At this time, HIF-1α provides a key braking mechanism in CFs by regulating mitochondrial ROS, preventing the over-activation and proliferation of CF after ischemia (Janbandhu et al., 2022). The results of this study confirm an abnormal increase in CFs proliferation, which is associated with higher levels of ROS.

### 3.5 Abnormal activation of inflammatory cytokines and inflammatory pathways

In type 2 diabetes mellitus (T2DM), the activation of inflammatory pathways not only leads to the formation of an inflammatory microenvironment inside the islet, which then damages the insulin secretion function of beta cells and causes insulin resistance, but also plays an important role in the occurrence and development of diabetic complications (Lontchi-Yimagou et al., 2013). For example, High-glucose increase the expression of inflammatory molecules such TNF-α, IL-1β, and IL-6, which can activate, proliferate, and migrate fibroblasts, leading to diabetic cardiac fibrosis (Zhao et al., 2024). The NF-κB signaling pathway is one of the most important transcription factors involved in proinflammatory gene expression. Its activation is a strong cause of myocardial inflammation in diabetes (Frati et al., 2017). Activating the NF-κB promotes CFs activation and fibrosis by increasing the production of pro-inflammatory genes such as TGF-β1 (Zou et al., 2022).

## 4 Inhibitory effect of Chinese herbal medicine on fibroblast activation

TCM demonstrates the potential to ameliorate DCM by inhibiting the activation of CFs under high-glucose conditions. Currently, an increasing number of studies are revealing the mechanisms by which Chinese herbs inhibit the activation of CFs, encompassing various aspects such as single Chinese medicinal herbs, prepared Chinese patent medicines, and complex TCM formulas. Table 1 details the key elements of Chinese medicine intervention experiments, while Table 2 systematically presents the specific composition of TCM compounds and proprietary Chinese medicines.

**TABLE 1:** The effect of Chinese medicine interventionon CFs activation experiments.

Category	Compounds	Intervention objects	Experiment model	Result	Molecular mechanism	Reference
Active ingredient in Chinese medicine	Allicin	HCFs	High glucose	HCFs proliferation↓, HCFs apoptosis↑	Bcl-2↑,Bax↓, Caspase −3↑	Xiaopei et al. (2023)
	Hesperetin	H9c2 cells	33 mmol/L D -glucose	Fbg↓,HbA1c↓, Inflammatory response↓, myocardial fibrosis↓, LVEF↑, LVFS↑, cardiac function↑	TNF-a↓, IL-1β↓ ICAM-1↓ VCAM-1↓ CollagenI↓ CollagenIII↓ α-SMA↓ NF-κB(P56)↓	Wanyuan (2017)
		CFs				
		SD rats	STZ 70 mg/kg			
	Gardenin	CFs	33.3 mmol/L glucose	fibroblast phenotypic transformation↓	Collagen I↓ Collagen III↓, TGF-β/ac-Smad3↓ α-SMA↓,SIRT1↑	Jiapei and Yuhua (2019)
	Ginsenoside Rg1	CFs	25 mmol/L D -glucose	myocardial fibroblast proliferatio↓	Collagen I↓ CollagenIII↓ TGF-β1↓ β-catenin↓ P-GSK-3β↓	Lutuo et al. (2014)
	Baicalin	H9c2 cells	35 mmol/L D -glucose	myocardial fibrosis↓	TGF-β1↓ Collagen I↓ Collagen III↓ p-Smad2/3↓	Kaijia et al. (2024)
		Human AC16 cells				
	Salvianolic acid B	SD rats	30 mg·kg-1STZ	myocardial fibrosis↓	α-SMA↓ Collagen I↓ Collagen III↓ RhoA↓ ROCK1↓	Xin and Lijuan (2022)
		CFs	25 mmol·L-1 glucose			
	Oxymateine	C57BL/6 rats	HFD + STZ 30 mg/kg	Fbg↓,HW/BW↓ TG↓,T-CHO↓ LDL-C↓ HDL-C↑ LVEF↑,LVFS↑, myocardial fibrosis↓ cardiac function↑	α-SMA↓ Collagen I↓ CollagenIII↓, fibronectin↓ SIRT1↑	Rongxiu et al. (2024)
	Icariin	Wistar rats	HFHG + STZ 30 mg/kg	Fbg↓,HW/BW CFs activation↓, inflammatory responses↓ myocardial fibrosis↓	TGF-β↓, c-Jun↓,p65↓ Ca2+ hyperactivities↓	Zhang et al. (2021a)
	Syringaresinol	Type1 diabetic mice	33 mmol/L D -glucose	LVEF↑,LVFS↑, myocardial apoptosis↓ myocardial fibrosis↓	IL-6↓,TNF-α↓ IL-1β↓,TGF-β↓, fibronectin↓ CK-MB↓, α-SMA↓ p-Smad2/3↓ Bcl-2↑,Bax↓	Li et al. (2020)
	Resveratrol	H9c2 cells	25 mmol·L-1 glucose	Fbg↓HW/BW, myocardial fibrosis↓ myocardial apoptosis↓ cardiomyocyte hypertrophy↓	IL-1β↓,IL-6↓ TNF-a↓,α-SMA↓ Collagen I↓ RAGE↓ NF-kB (p65)↓ TGF-β1/Smad3↓ CK-MB↓	Yanzhou (2023)
		SD rats	HFD + STZ 50 mg/kg/d			
	Ginkgolide B	SD rats	STZ 70 mg/kg	HW/BW↓, oxidative stress↓ myocardial fibrosis↓	TGF-β1↓ α-SMA↓, p-Smad2/3↓ MDA↓,SOD↑	Jiang et al. (2020)
TCM Compounds and proprietary Chinese medicines	Danzhi Jiangtang Capsule	SD rats	HFD + STZ 35 mg/kg	FPG↓, myocardial injury↓ myocardial fibrosis↓	Collagen I↓ CollagenIII↓ TGF-β1 TLR4↓,MyD88↓ NF-kB (p65)↓	Peipei (2020)
		CFs	30 mmol/L glucose			
	Xinyang Tablet	C57BL/6J rats	50 mg·kg-1 Pentobarbital Sodium	LVEF↑,LVFS↑, cardiac failure↓ myocardial fibrosis↓	AGEs↓ Vimentin↓ FSP1mRNA↓ CollagenI↓ CollagenIII↓, fibronectin↓ CTGF mRNA↓	Birong et al. (2023)
	Shensong Yangxin Capsule	Wistar rats	HFD + STZ 40 mg/kg/d	HW/BW↓ LVEF↑,LVFS↑, myocardial fibrosis↓ collagen deposition↓,cardi acfunctions↑	TGF-β1↓ Collagen I↓ CollagenIII↓ MMP-2↓ MMP-9↓ α-SMA↓ Smad7↑ p-Smad2/3↓	Shen et al. (2014)
	Xinshuaikang Capsule	SD rats	STZ 30 mg/kg	myocardial fibrosis↓ apoptosis↓ myocardial damage↓	TGF-β1↓, p-p38 MAPK↓ p-CREB↑ Bcl-2↑ Caspase-3↓	Xiaojin et al. (2024)
	Zuogui Jiangtang Shuxin Formula	MKR rats	HFD + STZ 40 mg/kg	inflammatory infiltration↓ collagen deposition↓ myocardial fibrosis↓	α-SMA↓ Collagen Ⅰ↓ Collagen Ⅲ↓ TNF-a↓,IL-1β↓ TLR4↓,NF-κB p65↓,p-NF-κB p65/NF-κB p65↓	Juan et al. (2024)
	Tongxinluo Capsule	SD rats	STZ 30 mg/kg	myocardial fibrosis↓ myocardial structure disturbance↓	TGF-β1↓	Wenrui et al. (2023)
	Wanjin-wenwu decoction	SD rats	HFHG + STZ 35 mg/kg	blood glucose↓ myocardial function↑ myocardial fibrosis↓	TNF-α↓,IL-6↓ ROS↓,MDA↓, p-AMPK↑ GF-β1↓ Collagen Ⅰ↓ CollagenⅢ↓	Shuang (2021)

**TABLE 2:** Composition of TCM compound formula.

Compound prescription of Chinese medicine	Traditional Chinese medicine composition
Danzhi Jiangtang Capsules	Cuscuta chinensis Lam., Alisma plantago-aquatica L., Paeonia × suffruticosa Andrews, Hirudo nipponica Whitman, Rehmannia glutinosa (Gaertn.) Libosch. ex DC., Pseudostellaria heterophylla (Miq.) Pax
Xinyang Tablet	Panax ginseng C. A. Mey., Epimedium brevicornu Maxim., Astragalus mongholicus Bunge, Leonurus japonicus Houtt., Ilex pubescens Hook. and Arn., Descurainia sophia (L.) Webb. Ex Prantl, Plantago asiatica L
Shensong Yangxin Capsule	Panax ginseng C. A. Mey., Ophiopogon japonicus (Thunb.) Ker Gawl., Cornus officinalis Siebold and Zucc., Salvia miltiorrhiza Bunge, Ziziphus jujuba Mill., Taxillus sutchuenensis (Lecomte) Danser, Paeonia lactiflora Pall., Eupolyphaga sinensis Walker, Nardostachys jatamansi (D. Don) DC., Coptis chinensis Franch., Schisandra chinensis (Turcz.) Baill., Os Draconis
Xinshuaikang Capsule	Codonopsis pilosula (Franch.) Nannf., Astragalus mongholicus Bunge, Cinnamomum cassia Presl, Polyrhachis dives F. Smith, Angelica sinensis (Oliv.) Diels, Angelica sinensis (Oliv.) Diels, Pheretima aspergillum (E.Perrier), Pyrola calliantha Andres, Achyranthes bidentata Blume
Zuogui Jiangtang Shuxin Formula	Panax ginseng C. A. Mey., Astragalus mongholicus Bunge, Ophiopogon japonicus (Thunb.) Ker Gawl., Cornus officinalis Siebold and Zucc., Rehmannia glutinosa (Gaertn.) Libosch. ex DC., Coptis chinensis Franch., Salvia miltiorrhiza Bunge, Pueraria montana var. Lobata (Willd.) Maesen and S.M.Almeida ex Sanjappa and Predeep, Crataegus pinnatifida Bunge
Tongxinluo Capsule	Panax ginseng C. A. Mey., Hirudo nipponica Whitman, Buthus martensii Karsch, Paeonia lactiflora Pall., Cryptotympanapustulata Fabricius, Eupolyphaga sinensis Walker, Scolopendra subspinipes mutilans L.Koch, Santalum album L., Dalbergia odorifera T. C Chen, Boswellia sacra Flück., Ziziphus jujuba Mill., Dryobalanops aromatica C.F.Gaertn
Wanjin-wenwu decoction	Pueraria montana var. Lobata (Willd.) Maesen and S.M.Almeida ex Sanjappa and Predeep, Pinus koraiensis Siebold and Zucc., Scutellaria baicalensis Georgi, Conioselinum anthriscoides (H.Boissieu) Pimenov and Kljuykov, Asparagus cochinchinensis (Lour.) Merr., Schisandra chinensis (Turcz.) Baill., Platycodon grandiflorus (Jacq.) A.DC., Angelica dahurica (Fisch. ex Hoffm.) Benth. and Hook. f. ex Franch. and Sav., Rheum officinale Baill., Actaea cimicifuga L., Raphanus raphanistrum subsp. Sativus (L.) Domin, Eupatorium japonicum Thunb

### 4.1 Effective components of Chinese medicine

#### 4.1.1 Allicin

Allicin (AC) is one category of organic sulfur compound extracted from the Allium sativum L., such as the head of garlic, from the genus allium of the allium family. In TCM, garlic is used for reducing swelling and treating dysentery. AC has been proved to have properties of anti-microbial, anti-tumor, anti-oxidation, among others (Xu et al., 2023). Xiaopei et al. (2023) found that HCFs showed increased proliferation in the high-glucose environment and that the anti-apoptotic gene Bcl-2 has higher expression. Instead, they noted a significant reduction in the proliferation levels of HCFs after AC (10 μg/mL and 20 μg/mL) intervention. One of the possible mechanisms may be associated with the activation of the mitochondrial apoptosis pathway by AC, which promotes the expression of the Bax protein, represses the expression of the Bcl-2 protein, and upregulates the expression of Caspase-3. The limitation of this study is that both Bcl-2 and Bax were only assessed at 72 h, and further research data should be added in the future.

#### 4.1.2 Hesperetin

Hesperidin (HP) is a chemical substance obtained by hydrolyzation of HP, and HP is widely found in rutaceae, including Citrus reticulata Blanco, Citrus aurantium L. and Citrus reticulata Blanco in the field of medicine (Salehi et al., 2022). In the field of medicine, HP has been used for the treatment and prevention of cancer, regulating blood lipids, anti-diabetes, and preventing cardiovascular diseases (Salehi et al., 2022). It has been found to inhibit cardiac remodeling through inhibiting PKCα/β II-Akt, JNK, and TGF-β1/Smad signaling pathways (Deng et al., 2013). A study by Wanyuan (2017) showed that HP can reduce the Fasting Plasma Glucose (FPG) and Glycated Hemoglobin A1c (HbA1c) levels in rats with diabetes induced by 70 mg/kg STZ. HP can also enhance the left ventricular ejection fraction (LVEF) and left ventricular fractional shortening (LVFS) in DCM rats with streptozotocin (STZ)-induced, thereby achieving the goal of improving cardiac function. HP significantly reduced the inflammation-induced injury of cardiomyocytes in high-glucose-induced H9C2 cells *in vitro* by suppressing the abnormal activation of NF-κB. Moreover, it can also alleviate the proliferation and differentiation of CFs induced by high glucose *in vitro* by inhibiting the excessive deposition of types I and III collagen, thereby achieving the goal of inhibiting MF. It also has an inhibitory effect on the activation of NF-κB in CFs. However, the experiment did not use NF-κB agonists and antagonists to further clarify the molecular mechanism by which HP regulates the proliferation, differentiation, and collagen synthesis capabilities of CFs under high-glucose conditions.

#### 4.1.3 Geniposide

Geniposide (GE) is an iridoid glycoside, which comes from the traditional Chinese medicinal *Plumeria rubra* L (Liu et al., 2007). The constituent has been reported to exert antipyretic, anti-inflammatory, analgesic, cardiovascular and cerebrovascular protective, lipid-lowering and hepatoprotective effects, inhibit insulin resistance, promote glucose metabolism and decrease glucose level (Dan et al., 2021; Liu et al., 2022). Xiang et al. (Jiapei and Yuhua, 2019) discussed the influence of GE on the CFs transformation and collagen synthesis in high-glucose-induced rats. Primary CF was extracted from newborn rats and stimulated with high glucose and intervened with 100 μmol/L GE. The results indicated that the mrna expressions of CollagenⅠandⅢ of rats in different concentrations of GE decreased after 24 h of stimulation with high glucose. With the increase of the concentration of GE, the inhibitory effect is enhanced. This result indicates that GE inhibited the phenotypic transformation of CF and collagen synthesis induced by high glucose in rats, and the mechanism may be related to the SIRT1-mediated TGF-β/ac-Smad3 signaling pathway and OS.

#### 4.1.4 Ginsenoside Rg1

Ginsenoside Rg1 (GRg1) is a kind of steroid saponin, which can be extracted from Panax ginseng C.A.Mey., Panax quinquefolius L., Kalanchoe pinnata (Lam.) Pers. and other Chinese herbs (Wu et al., 2013). GRg1 has extensive neurotrophic and neuroprotective effects. Meanwhile, many research studies indicate that GRg1 has potent pharmacological activity and is advantageous for a number of illnesses, such as diabetes (Alolga et al., 2020), obesity (Liu et al., 2018), hyperlipidemia (Wang T. et al., 2020) and non-alcoholic fatty liver (Xu et al., 2018). Han and colleagues (Lutuo et al., 2014) cultured the cardiac fibroblasts extracted from neonatal SD rats using 25 mmol/L glucose and intervened them with varying concentrations of GRg1 for 48 h. Then they found that found that 100 mg/L GRg1 could inhibit the proliferation of myocardial fibroblasts, reduce the secretion of collagen and TGF-β1 in myocardial fibroblasts cultured with hyperglucose, and inhibit the abnormal expression of Wnt signaling pathway, having the potential effect of anti-MF. Previous studies have indicated that the abnormal activation of the Wnt signaling pathway can lead to the occurrence of fibrotic diseases (Tao et al., 2016; Griffin et al., 2022).

#### 4.1.5 Baicalin

Baicalin is an active principle of the Scutellaria baicalensis Georgi and is a glucuronic acid conjugate. Baicalin and glucuronic acid are produced after hydrolysis, which have many functions such as clearing heat and detoxifying, anti-inflammatory, cholelitic, antihypertensive, diuretic and anti-allergic reaction (Xiping et al., 2003). It is involved in the regulation of immunity (Kim et al., 1999), anti-oxidation (Wei et al., 2015), anti-virus (Huang et al., 2017), anti-heart failure (Zhao et al., 2016). Kaijia et al. (2024) discovered through network pharmacology that multiple active components in Scutellaria baicalensis Georgi exert anti-DCM effects through multiple targets, and they focused on exploring the mechanism of the active component Baicalin in anti-DCM through cell experiments. They added different doses of Baicalin to rat H9c2 and human AC16 culture dishes cultured with 35 mmol/L glucose and found that the 10 μmol/L Baicalin group significantly inhibited the TGF-β1/Smad signaling pathway, improved MF, and played a protective role in myocardium. However, Baicalin has a direct impact on the differentiation of CFs still needs to be explored.

#### 4.1.6 Salvianolic acid B

Salvia miltiorrhiza Bunge’s primary water-soluble component, salvianolic acid B(SalB), is isolated from the plant’s roots and rhizomes (Xiyu et al., 2021). SalB has anti-fibrosis effect on tissues of various organs such as liver (Colca, 2020) and kidney (Xu et al., 2019), anti-tumor (Zhang et al., 2010), vascular endothelial cell protection (Ren et al., 2016) and anti-inflammation (Stumpf et al., 2013). Xin and Lijuan (2022) found that compared with the normal control group, the expression of α-SMA, Collagen I, Collagen III, RhoA and ROCK1 was abnormally increased in CFs isolated from neonatal SD mice treated with 25 mmol L^-1^ glucose. After pretreatment with SalB (25  μmol L^−1^) and ROCK1 inhibitor Y-27632 (10 μmol L^−1^), the viability of CFs was significantly inhibited, and the expression of the above proteins was significantly decreased. These results suggest that in the process of diabetic MF, high glucose can activate the RhoA/ROCK1 signaling pathway and increase the expression of collagen, thereby promoting the formation of MF. SalB has a protective effect on diabetic MF, and its mechanism may be related to the inhibition of RhoA/ROCK1 signaling pathway.

#### 4.1.7 Oxymateine

Oxymateine (OMT) extracted from the dried roots of Sophora flavescens Aiton, a plant in the Fabaceae family, is a naturally occurring active component. Modern pharmacological studies have indicated that OMT possesses a variety of pharmacological effects, including anti-fibrosis, anti-inflammatory, anti-tumor, anti-arrhythmia, and anti-virus, among which the protective effect on the heart is more significant (Lan et al., 2020). Currently, it can be used to treat blood-brain barrier injury caused by ischemia reperfusion (Jiao-Yan et al., 2021), viral hepatitis (He et al., 2016) and also play a role in lowering blood glucose and lipid (Guo et al., 2014). Ou et al. (Rongxiu et al., 2024) found that OMT(50 mg/kg) Significantly reduce the heart/body weight ratio (HW/BW), ameliorates myocardial hypertrophy, and improves cardiac function in DCM rats induced by a high-fat diet (HFD) combined with low-dose STZ. Additionally, through regulating lipid metabolism abnormalities and activating SIRT1 to modulate OS, OMT reduced the expression of proteins associated with MF under high-glucose conditions. Subsequent studies can complement *in vitro* cell experiments to more directly investigate the mechanisms by which OMT intervention inhibits the activation of CFs.

#### 4.1.8 Icariin

Icariin (ICA), the main active component of Epimedium sagittatum (Siebold and Zucc.) Maxim., belongs to 8-isopentenyl flavonoid glycosides, which can be extracted from the dried stems and leaves of various epimedium plants of the genus Epimedium, such as Epimedium Sagittarius and Epimedium pl (Zeng et al., 2022). It has antioxidant, anti-inflammation, and lipid adjustment activities among many others (Wang M. et al., 2020). Modern researches have shown that icariin has significant therapeutic effect on diabetes along with its complications (Jiang et al., 2024). Four potential target genes associated with T2DM (Jun, p65, NOS3, and PDE5A) were firstly bioinformatically screened by Zhang L. et al. (2021); then it was verified in animal experiments that ICA can treat high-fat-high-glucose (HFHG) diet combined with STZ-induced DCM rats, and the results showed that ICA improved blood sugar, HW/BW, intracellular Ca^2+^ hyperactivities and function in myocardium in DCM rats. Furthermore, by downregulating the p65/Jun signaling pathway to control inflammatory responses, ICA can ameliorate abnormalities in collagen metabolism, which is beneficial for the structural damage and MF in rats with T2DM.

#### 4.1.9 Syringaresinol

Syringaresinol (SYR) is a kind of plant lignan, which is one of the bioactive components present in the berries of Panax ginseng C.A.Mey. (Choi et al., 2021). It has the functions of anti-inflammatory (Bajpai et al., 2018), anticarcinogenic (Park et al., 2008), anti-aging (Choi et al., 2022), and alleviating diabetic retinopathy (Liu et al., 2024). Guangru et al. (Li et al., 2020) orally administred STZ-induced type 1 diabetes mice with SYR in every other day for 8 weeks, which significantly improves cardiac dysfunction and preventes cardiac hypertrophy and fibrosis. The anti-fibrosis mechanism of SYR may be related to the regulation of Keap1/Nrf2 and TGF-β/Smad signaling pathways. In addition, SYR can also regulate the key apoptotic proteins Bcl-2 and Bax to alleviate cardiomyocyte apoptosis. Regrettably, this experiment did not specify the dosage of SYP, and there is a lack of comparative effectiveness between different dosages.

#### 4.1.10 Resveratrol

Resveratrol (RES), a naturally occurring polyphenol with the chemical structure of 3,4′,5-trihydroxystilbene has been developed for a variety of cardiovascular diseases (Gal et al., 2021). The roots and rhizomes of Reynoutria japonica Houtt. are the primary parts used for the extraction of natural resveratrol (Zhang L. X. et al., 2021). It has extensive cardioprotective effects, which also raise the concern about its role in the prevention and treatment of DCM. Zhu et al. (Yanzhou, 2023) induced myocardial injury in rat and H9c2 cardiomyocytes with high-fat diet combined with STZ, finding that after continuous intervention with RES (100 mg/kg and 20 mg/kg) for 8 weeks, the level of myocardial fibroblast activation and fibrosis-related proteins (a-SMA and Collagen I) was obviously downregulated in high dose group. Its mechanism was that RES selectively acted on RAGE, with subsequent inhibition of the activation of high-glucose-induced NF-κB and TGF-β1/Smad pathways, participating in the myocardial injury treatment induced by high glucose in diabetes mellitus and MF.

#### 4.1.11 Ginkgolide B

Ginkgolide B (GB) is an extract of Ginkgo biloba L. of the ginkgo family, and it is currently recognized as the most potent antagonist of platelet-activating factor (Xia and Fang, 2007). GB can be used clinically for the treatment of cardiovascular diseases (Ye et al., 2024) and metastatic cancer (Yu et al., 2024). It also has a protective effect on damaged neurons (Liang et al., 2024), as well as anti-oxidation and anti-aging effects (Xia and Fang, 2007). Jiang et al. (2020) created a diabetic rat model by a single intraperitoneal injection of 70 mg/kg STZ, and then administered GB (5 mg/kg) continuously for 8 weeks. They found that the level of inflammatory cytokines in diabetic rats treated with GB was significantly reduced, the level of lipid peroxidation product (MDA) was decreased, the activity of superoxide dismutase (SOD) was enhanced, the antioxidant level of cardiomyocytes was increased, and OS was inhibited. In addition, GB alleviates cardiac fibrosis by reducing the expression of TGF-β1, α-SMA, p-Smad2 and p-Smad3 and inhibiting the activation of myocardial fibroblasts (Jiang et al., 2020). Nevertheless, the study lacks *in vitro* cell experiments for dual validation.

### 4.2 TCM compounds and proprietary Chinese medicines

#### 4.2.1 Danzhi Jiangtang Capsule

Danzhi Jiangtang Capsule (DJC) is a type of hospital preparation that invigorates qi and nourishes yin, promotes blood circulation, and removes blood stasis (Peipei, 2020). It was reported that Danzhi Jiangtang capsule could significantly lower the level of blood glucose and blood lipid in patients with type 2 diabetes, participate in treating DCM, and reduce the degree of MF (Sihai et al., 2024). Peipei (2020) studied the effect of DJC on the diabetic model rats induced by HFD combined with 35 mg/kg STZ and the proliferation model of neonatal SD rat-CFs cultured in 30 mmol/L glucose. He found that DJC could effectively reduce FPG and delay diabetes-induced myocardial damage. The mechanism is related to regulating TLR4-MyD88-NF-κB signaling pathway, inhibiting the expression of TGF-β1, Collagen I and Collagen Ⅲ protein induced by high glucose in CFs, and preventing diabetic MF However, due to the complexity of TCM components and the relative difficulty in the extraction process, the main components and specific mechanisms of TCM still need to be further studied.

#### 4.2.2 Xinyang Tablet

Xinyang Tablet (XYT) is a new compound Chinese patent medicine and have been approved for the treatment of heart failure (Hanqin et al., 2021). In the latest study in 2023, Birong et al. (2023) used Transverse aortic constriction (TAC) to establish a mouse model of heart failure, and the success of the model was confirmed by LVEF less than 50%. After that, different doses of XYT were used for 6 weeks. The results showed that compared with the model group, the LVEF and LVFS of the high, middle and low dose groups were increased, and the cardiac function of the mice was improved. In addition, the expressions of fibroblast markers (vimentin, Ferroptosis Suppressor Protein 1(FSP1) mRNA) and cardiac fibrosis-related markers (CollagenⅠ, Collagen Ⅲ, fibronectin, Connective Tissue Growth Factor (CTGF) mRNA) were decreased, and the cardiac fibrosis-area was significantly reduced. This may be achieved by XYT down-regulating the expression of AGEs and thereby inhibiting the activation of CFs. However, it remains to be verified whether XYT can address MF caused by excessive accumulation of AGEs in diabetes.

#### 4.2.3 Shensong Yangxin Capsule

Shensong Yangxin Capsule (SSYX) has been used for the treatment of arrhythmia and atrial fibrillation in Chinese clinic for a long time, and has the effects of invigorating qi and nourishing Yin, activating blood and clearing collaterals, and calming the heart (Hongwei et al., 2024). In order to investigate the effect of SSYX on MF in diabetic rats, Shen et al. (2014) adopted 40 mg/kg/d STZ combined with HFD to induce diabetic rats in experiment, and found that SSYX could alleviate MF and collagen deposition in T2DM rats, reduce the protein levels of TGF-β1 and p-Smad2/3 (marker of activation of the Smad signaling pathway), and increase the expression of Smad7 (an inhibitory regulator of TGF-β) in T2DM rats. There suggest that SSYX inhibits abnormal CFs activation by inhibiting TGF-β1/Smad signaling pathway, thereby improving fibrosis and cardiac function. The results indicate that SSYX may be one of the alternative drugs for the treatment of DCM.

#### 4.2.4 Xinshuaikang capsule

Xinshuaikang capsule (XSK) is an empirical formula summarized in clinical practice, which has the effects of invigorating spleen and benefiting kidney, removing blood stasis and benefiting water, and can protect diabetic kidney structure and kidney function (Yaping et al., 2023). Xiaojin et al. (2024), have demonstrated that XSK can inhibit the development of MF in diabetic rats induced by 30 mg/kg STZ, and its effects may be related to the regulation of the TGF-β1/p38 MAPK/CREB signaling pathway. The activation of the p38 MAPK signaling pathway can stimulate the expression of the transcription factor CREB, thereby promoting the production and activation of TGF-β1. Concurrently, the abnormal increase of TGF-β1 can also elevate the phosphorylation levels of p38 MAPK, further activating the p38 MAPK signal transduction pathway. However, XSK effectively inhibits the expression of TGF-β1 and phospho-p38 MAPK proteins, substantially improving MF in diabetic rats.

#### 4.2.5 Zuogui Jiangtang Shuxin Formula

Zuogui Jiangtang Shuxin Formula (ZJS) can nourish Yin and benefit Qi, promote blood circulation, and detoxify. In particular, which has the effect of alleviating myocardial damage (Xihua et al., 2011) and inhibiting myocardial hypertrophy (Xinyue et al., 2023) in diabetes mellitus. Juan et al. (2024) used 8-week-old male MKR mice as experimental subjects to establish a model of DCM with HFD combined with intrabitoneal injection of 40 mg/kg STZ. After 8 weeks of ZJS intervention, the pathological changes of myocardial tissue in mice were significantly improved. It was reported that ZJS could inhibit the TLR4/NF-κB pathway effectively, downregulate the expression level of inflammatory factors, α-SMA, CollagenⅠ and Ⅲ, reduce collagen deposition, inhibit myocardial inflammation and MF, which plays an important role in DCM intervention. Unfortunately, the optimal dosage of TCM and the targeted regulation mechanism are still unclear, which needs further research and analysis.

#### 4.2.6 Tongxinluo capsule

Tongxinluo capsule (TXL) has the effect of beneficial qi and blood circulation, Tongxinluo and pain relief. Clinical studies have shown that TXL can delay the progression of DCM and improve heart function in clinical treatment (Lihua et al., 2016). Studies have shown that the intervention of TXL with DCM can inhibit MF and improve myocardial pathological injury (Tiantian and Xiaomei, 2011). Wenrui et al. (2023), in order to find a new therapeutic target for MF, constructed a DCM rat model by one-time intrabitoneal injection of 2%STZ, and found that Tongxinluo capsule may improve MF and myocardial structural disorders in DCM rats by inhibiting the expression of TGF-β1 protein. Subsequent studies can focus on the molecular mechanism by which TXL regulates TGF-β1.

#### 4.2.7 Wanjin-wenwu decoction

Wanjin-wenwu Decoction (WWD) is a traditional Chinese medicinal formula that originates from the classic literature of the Korean ethnic group in China. It has the effect of tonifying the lung and strengthening the kidney, moistening the lung and promoting fluid development. Clinical studies have shown that WWD can improve myocardial damage in diabetes (Heying, 2018; Shuang, 2021). Yu and Haozhen (2023) believe that WWD can improve MF in DCM rats, which may be related to its regulation of the AMPK/TGF-β1 signaling pathway, antagonizing myocardial structural damage, and remodeling cardiac function. AMPK is a key enzyme in the process of energy regulation, capable of inhibiting the excessive accumulation of ROS and maintaining homeostasis. The activation of p-AMPK can suppress the expression of TGF-β1 and downstream factors Smad 2/3, thereby intervening in collagen synthesis and the differentiation of CFs. Experimental results show that WWD can significantly increase the phosphorylation level of AMPK and inhibit the expression of TGF-β1 protein, thus counteracting the MF damage induced by DCM.

## 5 Summary and prospect

DCM is a serious consequence of diabetes and a leading cause of death in diabetic people. MF is one of the key pathological links of DCM. Hyperglycemia activates CFs through multiple pathogenic responses, which is a critical link in the fibrosis process. Therefore, suppressing CFs from activating has become a focus of clinical research. Sitagliptin is to inhibit the abnormal activation of CFs by inhibiting TGF-β/Smad signaling pathway, so as to play an anti-fibrotic role and treat DCM as a common clinical drug. Chinese medicine has a long history in the treatment of diabetes, and in recent years, it has also shown its prominence in the prevention and treatment of DCM. Studies have proved that the active ingredients of CHM can indeed inhibit the activation of CFs and delay the process of MF by regulating pathological processes such as OS. This will undoubtedly provide a new Angle and target for future research on the treatment of DCM.

However, while collecting and summarizing the literature, I found that although the number of studies on the inhibition of cell activation by CHM to prevent and treat DCM is increasing, and most of them have reached a certain depth and gained recognition in the field, the fact is that some problems still exist, and a lot of research needs to be done in the future to fill the gaps. First of all, the research on the treatment of TCM by inhibiting CFs activation is still in the animal and cell experimental stage, lacking high-quality, multi-center, large sample, randomized, double-blind clinical trial data and evidence-based medical evidence to provide strong support. Secondly, most of the exploration of CHM still stays in extracts and proprietary Chinese medicines, and there is a lack of research on single CHM and Chinese drug pairs. Future research can focus on filling this gap. Finally, the multi-target and multi-pathway nature of CHMs determines that inhibition of cell activation involves more than one pathologic pathway. Subsequent research should actively use emerging technologies to improve the complexity and intersectionality of the mechanisms of action of CHM.

In conclusion, we anticipate that this review will prove the effectiveness and potential of CHM in both preventing and treating DCM, which will support the development of CHM as a novel drug for DCM therapy.
